# Single-cell and spatial omics in non-small cell lung cancer: dissecting metabolic reprogramming and tumor-immune ecosystems for precision pharmacology

**DOI:** 10.3389/fimmu.2026.1865367

**Published:** 2026-07-06

**Authors:** Zhenzhen Lian, Xu Jing Wu, Yangyang Zhou, Pengfei Tang, Honglei Ge

**Affiliations:** Department of Pharmacy, Yueqing Hospital of Wenzhou Medical University, Yueqing People's Hospital, Wenzhou, Zhejiang, China

**Keywords:** drug resistance, immunotherapy, metabolic reprogramming, non-small cell lung cancer, precision pharmacology, single-cell omics, spatial omics, tumor–immune ecosystem

## Abstract

Non-small cell lung cancer (NSCLC) is often presented as a success story for precision oncology, but that success remains uneven. Targeted agents and immune checkpoint inhibitors have changed treatment for molecularly defined or immune-responsive tumors, yet many patients relapse as resistant clones emerge, tumor cells shift state, and local immune pressure changes across the lesion. Bulk genomic and transcriptomic assays have been useful for clinical stratification, but they average signals across mixed cell populations and therefore miss rare resistant cells, transient drug-tolerant states, and spatially restricted tumor–immune interactions. Single-cell and spatial omics now provide a more direct way to examine these problems. In NSCLC, single-cell RNA sequencing, single-cell chromatin profiling, spatial transcriptomics, and multiplex imaging have begun to map malignant epithelial plasticity, persister-like states, dysfunctional immune compartments, stromal remodeling, and metabolically distinct niches. These approaches do not simply add resolution; they help connect cell state, metabolic activity, tissue location, and treatment response. This mini review discusses how single-cell and spatial omics are reshaping our understanding of metabolic reprogramming and tumor–immune ecosystems in NSCLC, and how these findings may inform biomarker discovery, patient stratification, resistance monitoring, and rational combination therapy.

## 1 Introduction

Non-small cell lung cancer (NSCLC) is the predominant form of lung cancer, a disease that remains one of the leading causes of cancer-related death worldwide (1, 2). Molecularly targeted therapies and PD-1/PD-L1 immune checkpoint blockade have reshaped the management of advanced NSCLC over the past two decades (2). Treatment selection now depends heavily on molecular and immune biomarkers, but many patients still relapse or fail to achieve durable disease control because tumors evolve through heterogeneity, adaptive drug resistance, lineage plasticity, immune escape, and metabolic rewiring (3). Tumors with the same driver alteration or similar PD-L1 expression can still behave very differently in the clinic. This mismatch between biomarker labels and clinical behavior makes NSCLC a useful setting for asking whether single-cell and spatial profiling can add information beyond mutation-based stratification. These approaches may be especially valuable when resistance is driven by cell-state shifts, metabolic dependencies, or spatially restricted tumor–immune interactions.

Metabolic reprogramming links malignant progression, immune evasion, and therapeutic resistance. NSCLC cells adjust glycolysis, oxidative phosphorylation, glutamine metabolism, lipid utilization, redox balance, and mitochondrial function in response to oncogenic signaling, hypoxia, nutrient limitation, and treatment pressure (4, 5). Tumor and immune cells also compete for nutrients and oxygen in the tumor microenvironment, while suppressive metabolites and local tissue stress reshape immune-cell metabolism (6). Because these processes differ across cell types and tissue regions, bulk transcriptomic or metabolomic approaches often miss their local organization.

Single-cell and spatial omics now allow NSCLC to be examined at the level of individual cells and their tissue context. This review focuses on studies that connect metabolic reprogramming with spatially organized tumor–immune ecosystems, and considers how these measurements may inform biomarker discovery, rational combination therapy, and individualized pharmacologic intervention. Rather than treating cell states, metabolic dependencies, and spatial immune architecture as separate catalogs, the review emphasizes where they converge on testable pharmacologic hypotheses in NSCLC.

## 2 Single-cell omics reveals cellular heterogeneity and metabolic reprogramming in NSCLC

### 2.1 Cellular heterogeneity and malignant cell states

**Figure 1:**
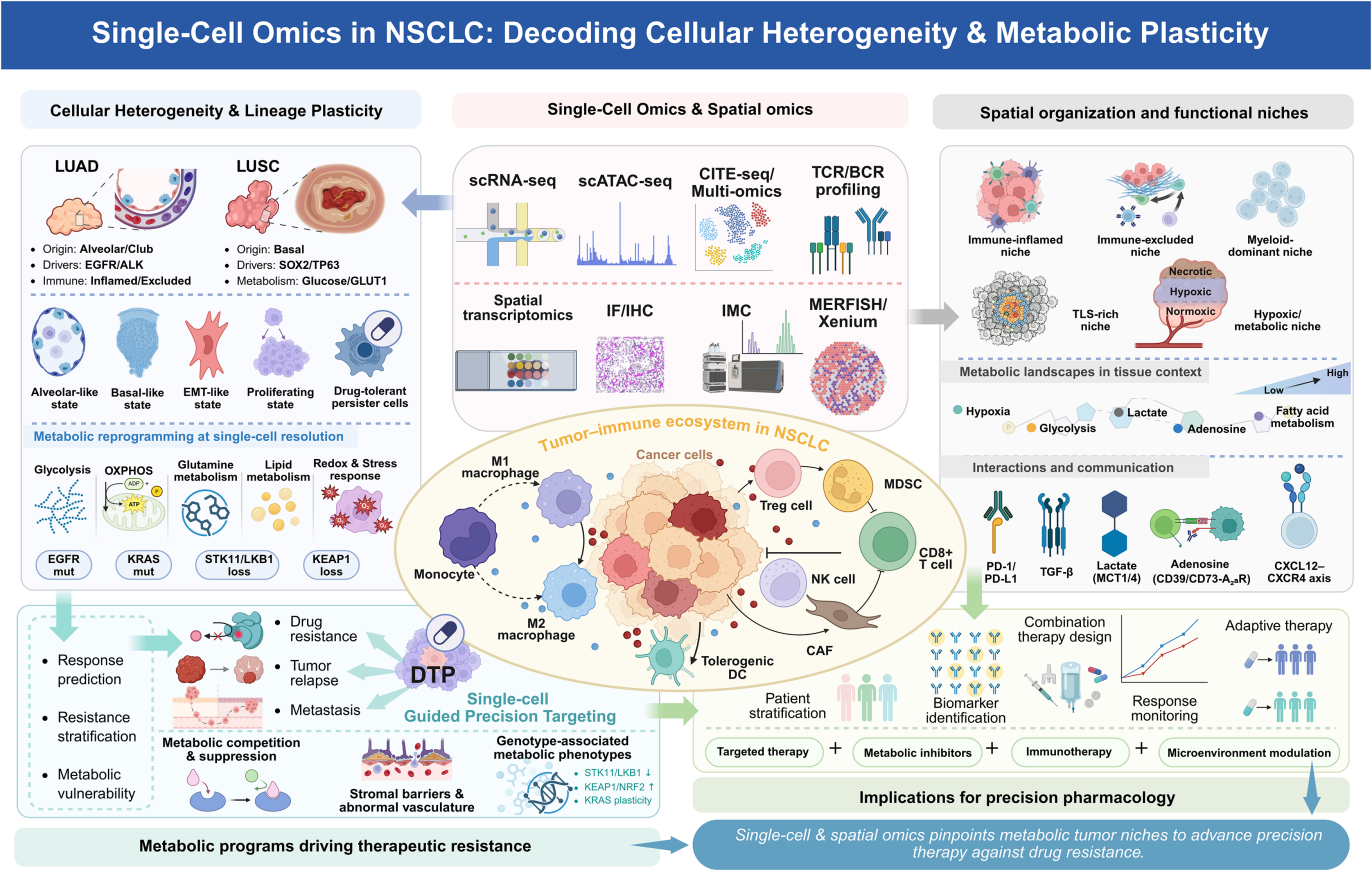
Single-cell and spatial omics reveal cellular heterogeneity, metabolic plasticity, and therapeutic resistance niches in NSCLC.

Single-cell omics has made the heterogeneity of NSCLC more explicit (**Figure 1**). Rather than appearing as uniform masses of malignant epithelial cells, tumors can be resolved into genetically, transcriptionally, and phenotypically diverse cell states. Lung adenocarcinoma and lung squamous cell carcinoma differ in lineage programs, differentiation trajectories, immune composition, stromal architecture, and therapeutic vulnerabilities. Within a single tumor, malignant cells may occupy states linked to proliferation, epithelial differentiation, epithelial-mesenchymal transition, stemness, senescence, hypoxia response, interferon signaling, and drug tolerance (7). By separating individual cells, single-cell RNA sequencing also helps identify rare subpopulations that may contribute to metastasis, relapse, or therapeutic resistance.

Several NSCLC studies illustrate how single-cell data can reconstruct malignant epithelial hierarchies and lineage plasticity. In lung adenocarcinoma, tumor cells may resemble alveolar type II-like, club-like, basal-like, or mixed transitional states, reflecting both cell-of-origin programs and ongoing tumor evolution. This plasticity is clinically important because treatment pressure may select resistant subclones that are already present or push cells toward drug-tolerant phenotypes (8). EGFR-mutant NSCLC, for example, may develop AXL-high tolerant programs that support survival before classic resistance mechanisms such as EGFR T790M or MET amplification emerge (9). KRAS-driven tumors, particularly those with STK11/LKB1 co-alteration, can show neutrophil-recruiting inflammatory programs that contribute to an immunosuppressive, T-cell-suppressed phenotype (10). EMT-like transitional states and shifts along the alveolar type II-to-basal or club-like lineage axis also mark cells with stem-like and treatment-tolerant properties (11). Lung adenocarcinoma (LUAD) and lung squamous cell carcinoma (LUSC) should therefore be separated where their biology diverges. LUAD is often associated with alveolar or club-like lineage programs and is enriched for actionable drivers such as EGFR and ALK (2), whereas immune responsiveness varies with smoking history, oncogenic driver status, and co-alterations such as STK11/LKB1 or KEAP1 (12, 13). LUSC more often retains basal or squamous differentiation programs, is commonly smoking-associated, has fewer routinely actionable single-driver alterations, and can show metabolic preferences including glucose and GLUT1 dependence (2, 14).

Single-cell ATAC sequencing and multi-omic approaches add a regulatory layer by linking cellular states to chromatin accessibility and transcriptional control. Chromatin accessibility profiling can reveal transcription factor networks that maintain malignant programs, including AP-1, MYC, HIF, YAP/TAZ, SOX factors, and lineage-specific regulators (15, 16). Single-cell omics also shows that therapeutic resistance is often not a purely tumor-intrinsic process. Resistant malignant states frequently coexist with suppressive macrophages, exhausted T cells, regulatory T cells, cancer-associated fibroblasts, and abnormal endothelial cells, suggesting that drug response emerges from coordinated tumor–microenvironment interactions (17, 18). For precision pharmacology, treatment selection may need to consider both oncogenic drivers and the cell states or local microenvironmental conditions that determine whether those drivers remain pharmacologically actionable (15).

### 2.2 Metabolic reprogramming at single-cell resolution

Metabolic reprogramming in NSCLC is heterogeneous and changes with oncogenic signaling, microenvironmental stress, and therapeutic pressure. Bulk studies have established the importance of glycolysis, oxidative phosphorylation, glutamine metabolism, lipid metabolism, nucleotide synthesis, and redox homeostasis in lung cancer, but single-cell studies show that these programs are unevenly distributed across malignant and non-malignant cell populations (5, 19). Within the same tumor, some malignant cells may carry glycolytic and hypoxia-associated signatures, whereas others show stronger mitochondrial respiration, fatty acid metabolism, or stress-response programs. This heterogeneity helps explain why inhibition of a single metabolic pathway often produces incomplete responses and why residual tumor cells may survive through metabolic compensation. The interpretation still requires caution. Most single-cell conclusions about glycolysis, oxidative phosphorylation, lipid metabolism, or hypoxia come from gene-set enrichment or module scores rather than direct measurement of metabolite levels or pathway flux, and these scores are sensitive to sequencing depth, dropout, normalization choices, and cell-cycle or stress-related confounders.

Oncogenic context strongly shapes metabolic states in NSCLC. EGFR signaling can influence glucose uptake, lipid synthesis, and mitochondrial function, while KRAS-driven tumors often display flexible nutrient utilization and dependence on stress-adaptation pathways. Foundational cancer-metabolism studies and lung-tumor metabolic tracing have established variable glycolytic, oxidative, glutamine, and lactate programs across tumors (4, 5). Direct isotope-tracing studies have also shown that lactate can fuel oxidative metabolism in human lung tumors (20). Alterations in STK11/LKB1 and KEAP1 are particularly important because they regulate energy sensing, oxidative stress responses, and immune-metabolic interactions (12, 21). Tumors with STK11 or KEAP1 alterations frequently show features of metabolic adaptation, redox remodeling, and resistance to immune checkpoint blockade, especially when co-occurring with KRAS mutations (12, 13). Single-cell analysis can help determine whether these phenotypes are uniform across all malignant cells or concentrated in specific resistant subpopulations. In addition, metabolic pathway inference from single-cell transcriptomes can nominate tumor-cell states with enhanced glutamine utilization, fatty acid oxidation, cholesterol metabolism, one-carbon metabolism, or mitochondrial biogenesis for functional validation (19).

Cell-state plasticity and therapeutic persistence are tightly linked to metabolic adaptation. Drug-tolerant persister cells may enter slow-cycling, EMT-like, or stress-adapted states that depend on oxidative phosphorylation, mitochondrial fitness, antioxidant defenses, ferroptosis-related vulnerabilities, or lipid metabolic programs (8, 22). Profiling tumors before treatment, during therapy, and at relapse may reveal metabolic adaptations before radiographic progression becomes apparent. Tumor metabolic programs also shape immune escape by consuming glucose and amino acids (23), producing lactate (24), or generating adenosine (25). Because these processes sit at the interface between malignant cells and the tumor microenvironment, single-cell metabolic inference is strongest when paired with spatial data and functional validation. NSCLC metabolism should therefore not be treated as a uniform cancer hallmark. It is better viewed as a set of cell-state-specific vulnerabilities that may support precision pharmacology. A related distinction is also necessary. Metabolic reprogramming refers here to tumor-intrinsic adaptation, in which malignant cells rewire glycolysis, oxidative phosphorylation, and nutrient utilization to support proliferation and survival under therapeutic pressure. Metabolic suppression refers to the impairment of immune-cell function caused by nutrient competition and inhibitory metabolites such as lactate, adenosine, and kynurenine. The two processes are connected, but they point to different therapeutic targets: tumor-intrinsic dependencies versus immune-cell metabolic dysfunction. We therefore distinguish tumor metabolic reprogramming in this section from immune metabolic suppression in Section 4.

## 3 Spatial omics maps tumor–immune ecosystems and metabolic niches

### 3.1 Spatial immune architecture

Spatial omics adds the tissue architecture that dissociation-based single-cell sequencing removes. In NSCLC, this architecture determines whether cytotoxic T cells contact tumor cells, whether macrophages form suppressive barriers, whether cancer-associated fibroblasts exclude lymphocytes, and whether metabolic stress is concentrated in particular tumor regions. Single-cell spatial profiling of lung tumors has also shown that immune-cell states form spatially distinct neighborhoods (26). Spatial transcriptomics (27), imaging mass cytometry (28), CODEX (29), MERFISH (30), seqFISH+ (31), and related high-plex *in situ* platforms can map molecular programs within intact tissue sections. Tertiary lymphoid structures, B-cell-rich aggregates, and dendritic cell niches further influence local immune surveillance and treatment response (32, 33). These spatial immune patterns should be interpreted with the technical limits of each platform in mind. Spot-based spatial transcriptomics methods, including standard Visium-style platforms, capture multicellular spots rather than individual cells, so each measurement may blend tumor, immune, and stromal signals and often requires computational deconvolution (34). Imaging-based methods can reach subcellular or single-cell resolution, but molecular coverage varies. MERFISH and many commercial *in situ* platforms usually rely on predefined gene panels, whereas seqFISH+ has demonstrated transcriptome-scale imaging across thousands of genes. Throughput, field of view, segmentation quality, and assay complexity all affect how confidently a study can assign a molecular program to a specific cell type or spatial niche (30, 31).

### 3.2 Spatial metabolic niches

Spatial profiling also places metabolic reprogramming within tissue ecology. Hypoxic and glycolytic tumor regions may localize near poorly perfused vessels, whereas lactate-rich or adenosine-producing niches may overlap with suppressive immune states (24, 25). Such neighborhoods could create local zones of immune dysfunction even when the tumor as a whole appears immune infiltrated. Integrating spatial transcriptomics with spatial proteomics can reveal functional neighborhoods that bulk profiling cannot resolve and that dissociated single-cell sequencing captures only indirectly (27, 35). For metabolic niche analysis, however, spatial transcriptomics and spatial proteomics remain indirect because they infer metabolic state from transcript or protein abundance rather than measuring metabolites themselves. Spatial metabolomics can address part of this gap. Mass spectrometry imaging, including matrix-assisted laser desorption/ionization (MALDI) imaging and related methods, can map small metabolites, drugs, and lipid species across tissue sections and display metabolic gradients more directly than transcript- or protein-based inference. Analyte coverage, metabolite identification confidence, sensitivity, and spatial resolution still depend on ionization method, matrix and sample preparation, instrumentation, and orthogonal validation (36).

### 3.3 Spatial pharmacology

From a pharmacologic perspective, these spatially defined ecosystems may help explain why tumors with similar driver mutations or PD-L1 levels respond differently to the same therapy. They may also point to combination strategies, such as pairing immune checkpoint blockade with metabolic inhibition, stromal remodeling, or other microenvironment-directed interventions, to shift excluded or suppressive niches toward more immune-permissive states. Spatial omics therefore adds a tissue-level layer to NSCLC profiling by linking cell types with functional neighborhoods that shape immune surveillance, metabolic competition, and pharmacologic response (34).

## 4 Tumor–immune metabolic ecosystems and therapeutic resistance

### 4.1 Immune-cell metabolism and immunosuppressive niches

The NSCLC immune microenvironment is metabolically organized. Immune dysfunction may reflect not only failed antigen recognition, but also nutrient competition, exposure to suppressive metabolites, and adaptation to hostile tissue conditions. Effector CD8+ T cells require glucose, amino acids, mitochondrial fitness, and balanced redox signaling to sustain proliferation, cytokine production, and cytotoxicity. In nutrient-depleted or hypoxic tumor regions, T cells may show reduced glycolytic capacity, impaired mitochondrial function, increased inhibitory receptor expression, and exhaustion-associated transcriptional programs (6, 23).

Regulatory T cells, macrophages, myeloid-derived suppressor cells, dendritic cells, NK cells, and B cells also undergo metabolic remodeling within NSCLC. Paired single-cell analyses of early lung adenocarcinoma have shown substantial remodeling of innate immune compartments, including macrophage and dendritic-cell states (37). Tregs can adapt to lactate-rich and lipid-rich environments, allowing them to preserve suppressive function in settings where effector T cells are metabolically constrained. Tumor-associated macrophages may acquire immunosuppressive phenotypes linked to fatty acid oxidation, cholesterol handling, arginine metabolism, oxidative stress responses, and hypoxia signaling (6, 38). These macrophages can suppress T-cell activity through IL-10, TGF-β, prostaglandins, reactive oxygen species, arginase activity, and checkpoint ligand expression. In contrast, dendritic cell subsets capable of cross-presentation are required for productive antitumor immunity, but they may be numerically reduced or functionally impaired in metabolically suppressive regions.

Tumor-derived metabolites help coordinate these immune states. Lactate accumulation can reduce cytotoxic T-cell and NK-cell function while promoting suppressive macrophage polarization and Treg stability (24, 38). Adenosine generated through CD39/CD73 activity inhibits effector lymphocytes and supports immune tolerance through A2A receptor signaling (25). Tryptophan catabolism through IDO and related enzymes produces kynurenine-pathway metabolites that impair T-cell responses and favor regulatory programs (39). Spatial omics can help show where these pathways are active and whether they overlap with immune-excluded or myeloid-rich regions. This spatial component matters because metabolic suppression is rarely uniform across the tumor. Thus, the tumor-immune ecosystem in NSCLC should be understood as a metabolically structured network in which nutrient competition and metabolite signaling shape the success or failure of immunotherapy (6). The strength of evidence for these mechanisms in NSCLC specifically varies and should not be overstated; much of the detailed mechanistic work still comes from other tumor types, *in vitro* systems, or murine models, and direct demonstration of metabolite-driven suppressive niches by single-cell or spatial profiling of human NSCLC remains comparatively limited.

### 4.2 Metabolic mechanisms of drug resistance and precision pharmacology

Therapeutic resistance in NSCLC is often attributed to secondary genetic alterations, but metabolic adaptation and cell-state plasticity may create drug-tolerant reservoirs before stable resistance mutations appear. This issue is particularly relevant for targeted therapies: EGFR inhibitors, ALK inhibitors, and KRAS G12C inhibitors can produce strong initial responses, yet relapse remains common (2). Drug-tolerant persister cells may enter slow-cycling or transcriptionally rewired states characterized by enhanced oxidative phosphorylation, mitochondrial dependence, antioxidant defenses, autophagy, lipid metabolism, or EMT-associated survival programs (22, 40). Standard genomic assays may miss these states, even though they influence whether residual tumor cells survive pharmacologic pressure. Single-cell profiling during therapy can detect persister populations earlier, while spatial omics may show whether they occupy protective niches enriched for fibroblasts, macrophages, hypoxia, or abnormal vasculature (8). Early detection of a persister state could help guide pharmacologic strategies. For example, a persister population dependent on oxidative phosphorylation or antioxidant defenses might support adding a metabolic or stress-adaptation intervention, such as targeting OXPHOS, GPX4/ferroptosis vulnerability, or autophagy, alongside the primary targeted therapy before stable resistance emerges (22). If persister cells are slow-cycling and re-expand after drug withdrawal, intermittent or adaptive dosing may help reduce the selective pressure that drives outgrowth. Serial monitoring, including circulating tumor DNA and other minimal residual disease readouts, could track whether such states expand and help time the introduction or switching of agents (41).

Metabolic resistance is also shaped by genotype-specific contexts. STK11/LKB1 loss disrupts energy sensing and can alter AMPK-mTOR signaling, immune infiltration, and metabolic stress adaptation. KEAP1 alterations activate NRF2-mediated antioxidant and detoxification programs, supporting redox balance under therapeutic and oxidative stress (12, 21). When STK11 or KEAP1 alterations occur with KRAS mutations, tumors may exhibit immune resistance, metabolic plasticity, and poor responses to immune checkpoint blockade. These features suggest that genomic biomarkers should be interpreted together with cell-state and metabolic phenotypes.

For NSCLC precision pharmacology, driver mutations alone are unlikely to be sufficient. Ecosystem-level biomarkers may help refine rational combinations, including targeted therapy plus metabolic inhibition, immune checkpoint blockade plus adenosine or IDO pathway blockade (25, 39), and immunotherapy-containing regimens whose benefit varies across biological contexts (12, 42). In EGFR-TKI-treated persister states, emerging evidence also supports metabolic cotargeting strategies directed at DPP4-associated fatty-acid uptake, oxidative phosphorylation, and antioxidant adaptation (43). These approaches may help distinguish pre-existing resistant subpopulations from therapy-induced adaptive states and refine treatment timing. Translation still requires functional validation, because transcriptomic metabolic signatures do not necessarily reflect metabolite abundance or pathway flux. Combining single-cell and spatial omics with metabolomics, ex vivo drug testing, organoid systems, and longitudinal biopsies may help bridge this gap (34, 44). The practical goal is not simply to match one mutation to one drug, but to target the evolving metabolic and immune ecosystems that sustain therapeutic resistance (**Table 1**).

**Table 1:** Summary of tumor–immune metabolic ecosystems and resistance mechanisms in NSCLC.

Category	Key metabolic features & components	Impact on therapeutic resistance	Potential therapeutic implication
Effector Immune Cells	Glucose/amino acid competition, mitochondrial fitness, redox signaling.	Nutrient depletion and hypoxia lead to T-cell exhaustion and dysfunctional states.	Support effector fitness: relieve nutrient/hypoxic stress; test ICB combinations with metabolic or anti-angiogenic strategies that may improve T-cell function.
Suppressive Immune Cells	Tregs: Lactate/lipid adaptation; TAMs: Fatty acid oxidation, arginine metabolism.	Preservation of suppressive niches; inhibition of T-cell activity via IL-10, TGF-β, and ROS.	Target suppressive programs: evaluate myeloid reprogramming, lipid/arginine metabolism blockade, or Treg-directed approaches alongside ICB.
Immunosuppressive Metabolites	Lactate accumulation, Adenosine (CD39/CD73), Kynurenine (IDO pathway).	Promotes myeloid polarization, inhibits NK/T-cell function, and supports immune tolerance.	Block metabolite axes: test lactate transport or pH-targeting strategies; CD39/CD73/A2A blockade; IDO/kynurenine-pathway inhibition.
Drug-Tolerant Persisters	Enhanced OXPHOS, antioxidant defenses, autophagy, and lipid metabolism.	Creation of metabolic reservoirs that survive targeted therapy (EGFR/ALK/KRAS inhibitors).	Test persistence-directed strategies: early metabolic blockade (e.g. OXPHOS, GPX4/ferroptosis, autophagy), adaptive/intermittent dosing, and MRD monitoring.
Genotype-Metabolic Links	*STK11/LKB1* loss (AMPK-mTOR disruption); *KEAP1* mutations (NRF2 activation).	Promotes redox balance and immune cold phenotypes, leading to poor ICB response.	Genotype-guided strategy: NRF2/glutaminolysis dependence in KEAP1; AMPK–mTOR and immune-modulating approaches in STK11-altered tumors.
Precision Pharmacology	Integration of spatial omics, metabolic inhibition, and cell-state plasticity monitoring.	Nomination of rational combinations (e.g., targeted therapy + metabolic blockade).	Ecosystem-informed combinations and treatment timing, requiring functional and clinical validation before application.

## 5 Discussion and future perspectives

Single-cell and spatial omics provide information that mutation-centered profiling alone does not capture: malignant cell states, metabolic dependencies, immune organization, stromal architecture, and spatially restricted drug-resistant niches (19, 45). Metabolic inference from transcriptomic data remains difficult because gene expression does not always correspond to metabolite abundance, enzyme activity, or pathway flux. Longitudinal sampling before treatment, during early pharmacologic response, at minimal residual disease, and after relapse will be important for mapping how NSCLC ecosystems evolve under therapeutic pressure. Future studies should test whether these measurements can define metabolic vulnerabilities, immune-ecological barriers, and adaptive resistance states that function as biomarkers for patient stratification, rational drug combinations, and longitudinal treatment adaptation (19, 34).

Clinical implementation remains a separate challenge. Biopsy timing and adequacy are limiting, because small diagnostic specimens may miss the resistant niche of interest and repeat sampling during therapy is often difficult. Tissue preservation is another constraint: many high-resolution assays require fresh or optimally fixed, rapidly processed material, whereas routine workflows still rely heavily on formalin-fixed, paraffin-embedded tissue. Cost and turnaround time are also poorly matched to treatment decisions in advanced disease, and analytical pipelines are not yet standardized, allowing results to vary across platforms and laboratories. These data must also be integrated with routine pathology and reported in a clinically interpretable form before they can support point-of-care decisions. Biopsy strategy, preservation protocols, cost and turnaround, computational standardization, and diagnostic workflow integration therefore need to be solved alongside biological validation (34, 45).

Overall, the value of single-cell and spatial omics in NSCLC lies in connecting defined cell states, metabolic dependencies, and spatial immune organization to pharmacologic hypotheses that can be tested in longitudinal, clinically annotated cohorts. Their outputs should be treated as hypothesis-generating and require functional and prospective validation before they change patient care.
